# Mutagen sensitivity and risk of second cancer in younger adults with head and neck squamous cell cancer: 15-year results

**DOI:** 10.1007/s00066-022-01917-2

**Published:** 2022-03-31

**Authors:** B. Bukovszky, J. Fodor, G. Székely, S. Zs. Kocsis, F. Oberna, T. Major, Z. Takácsi-Nagy, C. Polgár, Z. Jurányi

**Affiliations:** 1 Department of Oncology, Semmelweis University, Budapest, Hungary; 2 Department of Oral Diagnostics, Semmelweis University, Szentkirályi u. 47., Budapest, Hungary; 3 National Institute of Oncology, Budapest, Hungary

**Keywords:** Head and neck squamous cell cancer, Risk of second primary cancer, Survival with second primary cancer, Mutagen sensitivity, Bleomycin test

## Abstract

**Purpose:**

To evaluate the mutagen sensitivity phenotype on the risk of second primary cancer (SPC) in patients with head and neck squamous cell carcinoma (HNSCC), and to estimate the long-term rate of SPC and the outcome with SPC.

**Methods:**

A survey was made regarding SPC among 124 younger (≤ 50 years) adults with HNSCC who were enrolled in a pretreatment mutagen sensitivity investigation during 1996–2006. Mutagen sensitivity was assessed by exposing lymphocytes to bleomycin in vitro and quantifying the bleomycin-induced chromatid breaks per cell (b/c). Patients were classified as hypersensitive (> 1 b/c) or not hypersensitive (≤ 1 b/c).

**Results:**

Mean follow-up time for all patients was 68 months (range: 5–288 months), and the 15-year cancer-specific survival was 15%. Twenty patients (16%) developed a SPC (15-year estimated rate: 41%), and half of them was hypersensitive. The crude rate of SPC for hypersensitive (*n* = 65) or not hypersensitive (*n* = 59) patients were 15 and 17%, respectively (*p* = 0.4272). The 15-year estimated rate of SPC for hypersensitive and not hypersensitive patients was 36 and 48%, respectively (*p* = 0.3743). Gender, UICC stages, anatomical sites of index cancer did not prove to be a significant risk factor for SPC. Forty-five percent of SPC developed after the 10-year follow-up. The 3‑year cancer-specific survival was 23% with SPC.

**Conclusion:**

According to our findings, mutagen hypersensitivity was not associated with an increased SPC risk in HNSCC patients. Patients are at a lifelong risk of developing a SPC. Survival with SPC is very poor.

## Introduction

Smoking and excessive alcohol consumption are the main causal factors associated with HNSCC (head and neck squamous cell carcinoma). However, only a small proportion of smokers and drinkers (about 10%) develop HNSCC, suggesting that variations in genetic susceptibility may play an important role in the etiology of cancer [1, 2]. In 1983, the bleomycin assay was suggested by Hsu [3] as a biological marker for the development of environmentally induced cancer. The method is based on the scoring of bleomycin (BLM)-induced chromatid breaks occurring in cultured lymphocytes in vitro in the late G2 phase of the cell cycle. Spitz et al. [4] used this method to investigate the association between mutagen-induced chromosome damage and cancer risk and the interaction of carcinogenic exposures and chromosome damage in healthy and untreated patients with upper aerodigestive tract cancer. The cancer patients showed increased chromosome sensitivity (65.2% of cancer patients had > 0.8 b/c [breaks/cell]) compared to only 23.6% of the control patients had it, and chromosome sensitivity remained a strong and significant risk factor for head and neck cancer after adjustment for potential confounding from age, sex, cigarette smoking, and alcohol consumption. They concluded that the thesis that chromosome instability and/or defective DNA repair may underlie susceptibility to environmental carcinogenesis is plausible and may present a promising avenue for further research. Individuals with genetic instability may generate more cells with mutations or chromosomal aberrations than those with more stable genomes [5].

Young patients with HNSCC can be treated successfully with surgery and radiotherapy (RT), but they often develop second primary cancer (SPC). The leading cause of morbidity and mortality in these patients is the development of SPC [6]. Impact of smoking status, alcohol consumption, index tumor site, and disease stage on SPC development in patients with cancer of the oral cavity, pharynx, and larynx have been reported previously [7–9]. However, these factors do not account for the development of all SPCs. It is likely that genetic susceptibility also contributes to the development of SPC [10, 11]. Several studies have indicated that mutagen sensitivity may be a valuable biomarker of susceptibility to the development of multiple primary tumors [12–14].

The aim of this study was to determine the predictive value of mutagen sensitivity for the development of SPC in a group of HNSCC patients who were tested for mutagen sensitivity and treated between 1996 and 2006 at the National Institute of Budapest, Hungary [15, 16]. Another aim was to estimate the rate of SPC and the outcome with SPC.

## Materials and methods

Between 1996 and 2006, 432 patients with HNSCC underwent mutagen sensitivity assay before treatment. Informed consent was obtained from each study subject before enrollment and study protocol was approved by the institutional review board. The aim of that test was to clarify the usefulness of the bleomycin sensitivity assay elaborated in the USA as a biomarker of HNSCC, to explain the association between HNSCC susceptibility and exposure to carcinogens. The results were published elsewhere [15, 16]. From these patients, 124 met the following conditions: drinker and smoker, age ≤ 50 years at the time of bleomycin test, human papillomavirus (HPV)-negative oral cavity, pharyngeal (except nasopharyngeal) or laryngeal squamous cell cancer, treatment and follow-up were performed at our institute. We reviewed records of patients and compared pretreatment mutagen sensitivity between patients with or without SPC. SPC was partly defined according to the criteria of second primary tumor prevention trial of the M.D. Anderson Cancer Center [11]: the SPC must be diagnosed as malignant, has to be at least 2 cm from site of the index tumor, and has to occur ≥ 4 months after the diagnoses of the index tumor. Mutagen sensitivity was measured in vitro in lymphocytes by counting chromatid breaks induced by bleomycin as described previously [15, 16]. Briefly, blood cultures were incubated for 3 days and then exposed to bleomycin (30 µg/ml) for 5 h. Cells were harvested, and chromatid breaks were scored in 100 metaphases per sample, and recorded as the mean number of breaks per cell (b/c). The patient was classified hypersensitive if the mean number of b/c was > 1. The following survival endpoints were used: any death for overall survival, death from head and neck cancer for cancer-specific survival, death from SPC for survival with SPC, the appearance of SPC for SPC-free survival (time to SPC). Intervals to endpoints were examined with Kaplan–Meier method [17], and the curves were compared with log-rank test. The effect of the possible prognostic factors on the probability of the incidence of a SPC were examined in Cox regression model [18]. Statistical differences in proportions and means were assessed by 2‑sample t‑test and by Fisher exact test. GraphPad Prism (version 5.01 for Windows, GraphPad Software, San Diego, CA, USA) and Statistica (version 13.5.0.17, TIBCO Software Inc., Palo Alto, CA, USA) program packages were used for data analysis. A *p* value ≤ 0.05 was considered statistically significant.

## Results

Cytogenetic and clinical characteristics of the patients are given in Table 1. Of the four anatomical sites, tumors of oral cavity (34%) represented the largest group. The rate of patients with stage IV disease was high (43%), and few patients (*n* = 12) were treated with chemotherapy alone. Most patients were subjected to surgery and adjuvant RT. Therapy is detailed as follows: 86 patients were subjected to surgery. The number of R0 or R1 resection was 61 and 25, respectively. Of the 86 patients, 84 underwent lymphadenectomy: 20 were node negative and 64 were node positive. Extracapsular tumor extension (ECE) was seen in 14 node-positive patients. Response rate (complete or partial response) for radiochemotherapy alone, definitive RT or chemotherapy alone, was 9/10, 6/7 and 7/12.

**Table 1:** Clinical and cytogenetic characteristics

Characteristics	Patients, *n* (%)
*Mean age*	
45.8 years (range: 23–50 years)	124 (100)
*Mutagen sensitivity*	
Hypersensitive (> 1 b/c)	65 (52)
No hypersensitive (≤ 1 b/c)	59 (48)
*Anatomical subsite*	
Oral cavity	48 (38)
Oropharynx	27 (22)
Hypopharynx	32 (26)
Larynx	17 (14)
*Gender*	
Male	107 (86)
Female	17 (14)
*UICC stage*^a^	
I	3 (2)
II	20 (16)
III	48 (39)
IV	53 (43)
*Treatment*^b^	
Surgery alone	9 (7)
Radiotherapy alone	7 (6)
Surgery + adjuvant radiotherapy	71 (57)
Surgery + radiochemotherapy	6 (5)
Radiochemotherapy	10 (8)
Chemotherapy alone	12 (10)
Palliative therapy	9 (7)

*b/c* chromatid breaks/cell

^a^UICC Union Internationale contre de cancer, TNM Classification of Malignant Tumours—7th edition

^b^Treatments are detailed in the text

Mean follow-up time for all patients and alive patients was 68 months (range: 5–288 months) and 222 months (range: 184–249 months), respectively. Nine patients are still alive, and 115 have died. Crude overall survival rate is 7%. Ten patients died of internal disease. The crude rate of cancer-specific survival is 15%. The estimated rate of 15-year overall or cancer-specific survival is 14.5 and 19%, respectively.

Out of 124 patients, 20 (16.1%) developed SPC. The characteristics of the 20 patients are given in Table 2. The majority (*n* = 13; 65%) of SPC were HNSCC. Seven of them developed outside of head and neck region (esophagus, lung, prostate). The mean time to SPC was 118 months (range: 4–272 months). The 10-, 15-, or 20-year estimated rate of SPC was 24, 41 and 65%, respectively. In 3 patients, the index cancer and the SPC occurred in the same subregion (oral cavity, contralateral edge of the tongue). In these 3 cases the time to SPC was 161, 99 and 152 months (more than 5 years), and in every case the distance between the index cancer and SPC was greater than 2 cm. None of the patients with SPC had persistent disease or *percontinuitatem* invasion associated with SPC. The therapy of index cancer for patients with SPC was as follows: surgery alone (*n* = 2, R0 resection), surgery and adjuvant RT (*n* = 17, R0 or R1 resection 16 and 1), surgery and adjuvant radiochemotherapy (*n* = 1, with R1 resection and extracapsular tumor extension).

**Table 2:** Characteristics of the 20 primary cancer patients

	Gender	Index cancer	b/c	UICC stage	Site of SPC	UICC stage	Time to SPC (months)	Histology	Survival with SPC (months)
1	Male	Oral cavity	0.83	II	Oropharynx	II	109	SCC	31
2	Male	Oral cavity	0.97	I	Oral cavity	III	161	SCC	15
3	Male	Oral cavity	1.58	II	Esophagus	III	165	SCC	15
4	Male	Oral cavity	1.27	II	Oral cavity	III	99	SCC	82
5	Male	Hypopharynx	1.02	III	Oral cavity	III	24	SCC	30
6	Male	Hypopharynx	0.59	III	Lung	III	36	SCC	14
7	Male	Hypopharynx	1.51	III	Esophagus	III	24	SCC	10
8	Male	Larynx	0.55	II	Oropharynx	IV	208	SCC	10
9	Male	Larynx	0.64	II	Oropharynx	III	84	SCC	12
10	Male	Larynx	0.52	III	Oropharynx	IV	170	SCC	13
11	Male	Larynx	0.87	III	Oral cavity	IV	236	SCC	8
12	Male	Oral cavity	0.88	II	Lung	III	100	SCC	11
13	Male	Oral cavity	0.72	III	Esophagus	III	73	SCC	8
14	Male	Larynx	0.78	III	Lung	III	15	SCLC	12
15	Male	Oral cavity	1.52	II	Oral cavity	III	152	SCC	48
16	Male	Oropharynx	1.14	III	Oral cavity	III	4	SCC	81
17	Female	Larynx	1.34	III	Oropharynx	III	156	SCC	16
18	Female	Oral cavity	1.60	II	Larynx	III	74	SCC	12
19	Female	Hypopharynx	1.05	III	Oropharynx	II	235	SCC	10
20	Male	Oropharynx	1.45	II	Prostate	III	272	AC	16^a^
Mean	–	–	1.02	–	–	–	118	–	22

*b/c* chromatid breaks/cell, *UICC* Union Internationale contre de cancer, *TNM* Classification of Malignant Tumours 7th edition, *SPC* Second primary cancer, *SCC* squamous cell cancer, *SCLC* small cell lung cancer, *AC* adenocarcinoma

^a^19 patients died of cancer and 1 died of coronary disease

**Fig. 1:**
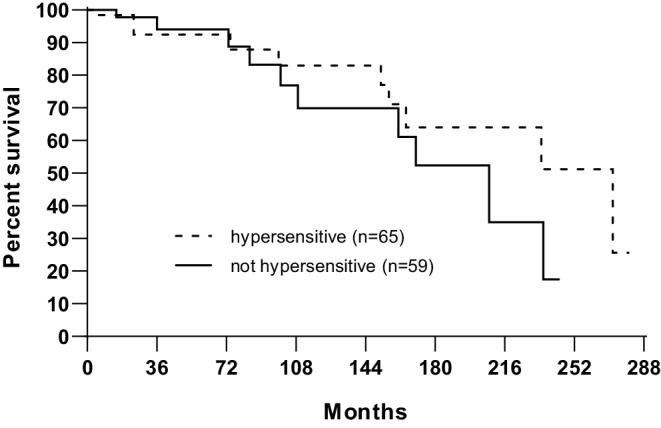
Second cancer-free survival by mutagen sensitivity. The 15-year estimated rate of second primary cancer of not hypersensitive or hypersensitive patients was 48 and 36%, respectively (*p* = 0.3743)

**Fig. 2:**
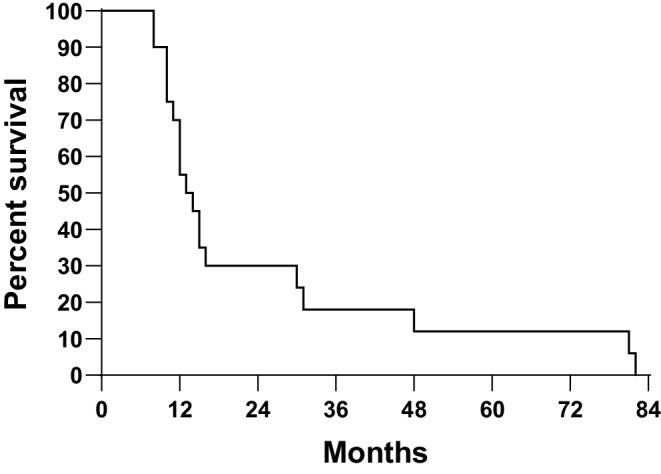
Cancer-specific survival with second primary cancer (*n* = 20). The estimated rate of 2- or 3‑year survival was 38 and 23%, respectively

The number of hypersensitive (> 1 b/c) patients were 65 (mean b/c: 1.43 ± 0.39). Ten of them (15%) developed SPC. In the not hypersensitive group (*n* = 59, mean b/c: 0.74 ± 0.18), 10 patients (17%) also developed SPC (*p* = 0.4272). The mean value of b/c for patients with SPC and without SPC was 1.02 ± 0.37 (range: 0.52–1.6) and 1.12 ± 0.48 (range: 0.35–2.8; *p* = 0.4062). The mean value of b/c was separately evaluated for patients with ≥ 36 months to SPC development. The mean b/c value of patient with “late” (≥ 36 months) SPC (*n* = 16) or patients without SPC (*n* = 104) was 1.02 (range: 0.52–1.60) and 1.12 (range: 0.35–2.80), respectively (*p* = 0.5724). The 15-year estimated rate of overall survival for hypersensitive or not hypersensitive patients was 16.9 and 11.0%, respectively (*p* = 0.4164). The second cancer-free survival curves by mutagen sensitivity are shown in Fig. 1. The 15-year estimated rate of second primary cancer for not hypersensitive and hypersensitive patients was 48 and 36%, respectively (*p* = 0.3743). The median and mean survival time with SPC was 23 months (range: 8–82 months) and 15 months. The 2‑ and 3‑year cancer-specific survival with SPC was 38 and 23%, respectively (Fig. 2). The 45% of SPC was developed after 10 years (between 152 and 272 months). The crude rate of SPC for men and women was 16% (17/107) and 18% (3/17), respectively (*p* = 0.9999), and for irradiated and nonirradiated patients 18% (18/94) and 7% (2/30), respectively (0.1540). It should be noted that 80% (*n* = 24) of the non-irradiated patients had stage IV disease. The rate of SPC by anatomical site of index cancer and UICC stage is given in Tables 3 and 4. The crude rate of SPC was significantly higher among patients with limited disease. However, the majority (62%) of our patients had stage III–IV disease and disease stage had a significant impact on cancer-specific survival. The 15-year cancer-specific survival with stage I, II, III or IV disease was 67, 52, 22 and 0%, respectively (multigroup *p* < 0.0001). All of the patients with stage IV disease died within 77 months. The short survival time might be one of the reasons that none of these patients developed SPC. The effect of the individual patient characteristics (gender, index cancer site, UICC stage, mutagen sensitivity, RT) on the risk of SPC was also examined in Cox proportional hazards model. Results are presented in Table 5. None of the studied variables proved to be a significant predictor of the risk of SPC.

**Table 3:** Second primary cancer rate by index cancer site

Index cancer	Patients, *n* (%)	b/c, mean ± SD	Mean FUP time^a^, months (range)	Crude rate, % (*n*)
Oral cavity	48 (38)	1.18 ± 0.51	70 (5–240)	13 (6)
Oropharynx	27 (22)	1.10 ± 0.42	75 (6–288)	15 (4)
Hypopharynx	32 (26)	1.10 ± 0.50	49 (6–244)	13 (4)
Larynx	17 (14)	0.90 ± 0.28	84 (7–244)	35 (6)
All	124 (100)	1.10 ± 0.47	68 (5–288)	16 (20)

*b/c* chromatid-break/cell, *SD* standard deviation

^*a*^*FUP time* follow-up time; Oral cavity + Oropharynx vs*.* Hypopharynx + Larynx *p* = 0.6253

**Table 4:** Second primary cancer rate by UICC stage

Stage	Patients, *n* (%)	b/c, mean ± SD	Mean FUP time^a^, months (range)	Crude rate, % (*n*)
I	3 (2)	1.09 ± 0.42	216(176–240)	33 (1)
II	20 (16)	1.20 ± 0.40	145 (21–288)	45 (9)
III	48 (39)	1.01 ± 0.48	82 (11–249)	21 (10)
IV	53 (43)	1.15 ± 0.47	17 (5–77)	0 (0)
All	124 (100)	1.10 ± 0.47	68 (5–288)	16 (20)

*b/c* chromatid breaks/cell, *SD* standard deviation, *Stage* TNM Classification of Malignant Tumours 7th edition

^*a*^*FUP time* follow-up time; stage I–II vs*.* stage III–IV *p* = 0.0009

**Table 5:** The 15-year estimated rate of second primary cancer by variables (univariate analysis)

Variables	%	*p*-value	RR (95% CI)
*Gender*	–	0.5071	–
Male	46	–	1.00
Female	24	–	0.6698 (0.2277–1.970)
*Site of index cancer*	–	0.1049	–
Oral cavity + oropharynx	39.5	–	1.00
Hypopharynx + larynx	44.1	–	2.009 (0.7924–5.084)
*UICC stage*	–	0.9615	–
Early (I + II)	46.4	–	1.00
Locally advanced (III–IV)	31.9	–	0.9797 (0.4078–2.354)
*Mutagen sensitivity*	–	0.3072	–
Not hypersensitive	47.6	–	1.00
Hypersensitive	36.1	–	0.6463 (0.2634–1.586)
*Radiotherapy*	–	0.8767	–
No	28	–	1.00
Yes	43	–	1.120 (0.2427–5.172)

*RR* relative risk, *CI* confidence interval, *UICC stage* TNM Classification of Malignant Tumours 7th edition

## Discussion

Several studies reported that bleomycin-induced mutagen sensitivity, reflecting latent genetic instability, is a significant predictor of SPC. HNSCC patients with SPC have higher b/c scores than SPC-free individuals [12–14, 19–21]. Schantz et al. [12] from the M. D. Anderson Cancer Center (Houston) used the bleomycin test first to estimate the risk of SPC in patients with HNSCC. The rate of SPC was significantly higher in the hypersensitive (b/c > 1) than in the not hypersensitive group (33% vs*.* 8%), and hypersensitivity increased the risk by 4.4 times. A weakness of their study was the short follow-up time: median < 2 years (range, 4–31 months). They extended the study to include 278 patients, and the results were published in 1994. Mutagen hypersensitivity (b/c > 1) also increased the risk of developing SPC. The mean b/c value for patients with SPC was 1.17 (±0.54) compared with 0.98 (±0.44) for SPC-free patients (*p* = 0.044). The follow-up time was again very short. The mean time from the diagnosis to the development of the SPC was only 10.5 months [13]. The mean time to SPC for our patients was more than 10 times longer (118 months). Cloos et al. [19] examined the mutagen sensitivity as a biomarker of SPC in Dutch HNSCC patients (*n* = 218). Nineteen of them (8.7%) developed SPC. The follow-up time was relatively short (median: 4.5 years). In this prospective study there was no difference between the groups (with or without SPC) with respect to mutagen sensitivity. They also found that patients who developed SPC ≥ 3 years after the index cancer had a significantly higher mean b/c value compared to patients with early SPC. In our study, the mean b/c value of patient with “late” (≥ 36 months) SPC (*n* = 16) or without SPC (*n* = 104) was not significantly different. Minard et al., from the M. D. Anderson Cancer Center studied the risk of SPC in patients with early stage HNSCC, who were enrolled in a placebo-controlled chemoprevention trial of low-dose 13-*cis*-retinoic acid to reduce the occurrence of SPC. Of the 1080 participants, a sample of 303 Caucasian patients was potentially available for their analysis. Overall, 50 of 303 patients (16.5%) developed SPC. Data on average follow-up time were not provided. The bleomycin-induced chromatid breaks were not associated with an increased risk. The lack of association between mutagen hypersensitivity and SPC was partly attributed to early UICC stage (I–II) of the enrolled patients [20]. A half year later, they published their experience with 991 patients. The mean follow-up time for living patients was 7 years, and the b/c cutoff value for mutagen sensitivity was 0.50. Among patients with SPC, the hypersensitive (b/c ≥ 0.5) and not hypersensitive (b/c < 0.5) rate was 82% (*n* = 243) and 18% (*n* = 55), respectively, *p* = 0.036 [21].

Our patients with HNSCC before treatment underwent bleomycin sensitivity assay and the method was not suitable for the assessment of individual index cancer risk due to overlapping of b/c values with those of controls [15, 16]. Here, we present our experience with the association between SPC and mutagen sensitivity in younger adults with HNSCC with the longest published follow-up time: mean time for all patients and alive patients was 5.8 and 18.5 years, respectively. The long follow-up time has to be emphasized, as patients are at high risk of developing SPC even 10 years after initial treatment. In our series, 45% of SPC developed after 10 years. In the current analysis, mutagen hypersensitivity was not significantly associated with an increased risk of developing SPC. The 15-year estimated rate of SPC for hypersensitive (b/c > 1.0) and not hypersensitive (b/c ≤ 1.0) patients was 36 and 48%, respectively. Cloos et al. [14] found significantly higher break/cell values in patients with multiple cancer (mean, 1.2), than in patients with a single cancer (mean, 0.96; *p* = 0.025). Our comparable values were 1.02 and 1.10 (*p* = 0.4062), respectively. The investigators from the M. D. Anderson Cancer Center changed the experimental parameters several times. First, the cutoff value for b/c was 1.0, and later it was decreased to 0.5. Furthermore, in their large-scale study, the SPC was counted together with local relapse [21]. In our series, the number of patients with b/c ≤ 0.5 was only nine and local relapse was not included in the analysis. Metastasis or local relapse was not considered as SPC.

Several studies investigated the risk of SPC and its impact on survival independently of mutagen sensitivity, and published long-term (more then 10-year) results [22–24]. In a large multicentric study (99,257 patients), the 20-year cumulative rate of SPC was 36%, and smoking and alcohol drinking increased the risk of developing SPC. Increased risks of SPCs persisted 10 years after diagnosis of the first primary [22]. Our patients also had a lifelong risk of developing SPC. All of our patients were drinkers and smokers, and the majority of them did not stop smoking and drinking. Otolaryngologists from Oslo studied 2063 head and neck patients [23]. The crude rate of SPC was 17%. The mean time to SPC was more than 4 years, and the median survival time with SPC was 12 months. SPC was most common in patients with limited (stage I/II) disease. Patients with a poor prognosis did not live long enough to develop SPC. In our series, none of the patients with stage IV disease developed SPC, and the crude rate of SPC was 16%, close to their rate. The survival with SPC was also very poor. The median and mean survival time was 23 and 15 months, respectively. In the study of Tiwana et al. from British Columbia, Canada [24], the follow-up time was 25 years (median time for alive patients: 23.2 years), and the crude incidence of SPC was 27%. Oral cavity and oropharyngeal index cancer were more likely to develop SPC. The estimated 5‑year overall survival with SPC was 15%. According to our analysis the anatomical subsite had no significant impact on the risk of SPC. In a German observation study [25], 118 patients (close to our sample) with HNSCC were selected in a SPC survey. The crude rate of SPC was 18%. Interestingly, 52% of SPC were diagnosed within 2 years. At our patients, only 2 SPCs were diagnosed within 2 years (4 and 15 months). Bugter et al. [26] from the Erasmus Cancer Institute, Rotterdam studied the risk factor of SPC in a Cox model. The crude rate of SPC was 15.6%. Smoking and alcohol consumption, comorbidity, and the oral cavity subsite were risk factors for SPCs. All of our patients were drinkers and smokers; therefore, we did not investigate these potential risk factors. In a current, population-based study from the United States, the smoking-related cancers were studied. Among 10 smoking-related cancer sites head and neck cancer patients had the highest risk of developing a SPC [27]. We studied the following risk factors of SPC in a Cox model: gender, UICC stage, site of index cancer, RT, and mutagen sensitivity (all of our patients were smoker and drinker). None of them proved to be a significant predictor of SPC development. Arie et al. [28] from Israel assessed the incidence of SPCs in patients with head and neck malignancies, according to treatment modality. Neither RT nor chemotherapy was associated with SPC development. Patients with an advanced-stage cancer had less time to develop SPC compared to early stage patients. Lifelong follow-up has to be emphasized because of permanent risk of SPC. Boakye et al. [29] examined the incidence and sites of SPCs stratified by a first HPV-associated HNSCC compared with non-HPV-associated HNSCC. Incidence of SPCs was higher among those with non-HPV-associated HNSCC than from potentially HPV-associated HNSCC. Among 109,512 patients with first HNSCC, 13,517 (12.3%) developed SPC (9.6% for patients diagnosed with a first potentially HPV-associated HNSCC and 14.0% for patients with a first non-HPV-associated HNSCC). All of our patients had HPV-negative cancer.

Chemoprevention of SPC in HNSCC patients are discussed in several publications [4, 5, 12, 19, 21]. In a large multicentric trial from the United States, the conclusion was that further prevention trials are needed to find an appropriate compound. In this trial, the low-dose 13-isoretinoic acid for 2 years did not decrease the incidence of SPC. Mutagen sensitivity as a biomarker was not involved in this study to define patients with high risk of SPC development [30]. Earlier, the same compound was used in the MD Anderson chemoprevention trial [20, 21]. Bhatia et al. stated: “Our findings did not confirm findings from the pivotal MD Anderson trial that used high-dose, short-term isotretinoin in patients with stage I–IV HNSCC. There were no statistically significant benefits in either OS or SPT” [30]. According to the published data, in clinical practice we have no appropriate biomarker to define risk of SPC development or compound to prevent its development in patients with HNSCC. Both former and current publications [31, 32] suggest long-term follow-up and early detection to improve outcome of patients with SPC. Patients (distinctively smokers and drinkers) have lifelong risk of SPC development.

## Conclusion

HNSCC survivors had an increased lifelong risk of developing SPC. The risk of developing SPC was higher in patients with less advanced cancer. Its incidence rate is high even after the 10-year follow-up. Survival is poor with SPC. Our results show that mutagen hypersensitivity does not increase the risk of SPC development. Therefore, mutagen sensitivity cannot be used as a biomarker to predict which patients will develop SPC.
